# The Sentient Cell: Implications for Osteopathic Medicine

**DOI:** 10.7759/cureus.54513

**Published:** 2024-02-20

**Authors:** Bruno Bordoni, Allan R Escher, Fabio Castellini, Joanna Vale

**Affiliations:** 1 Physical Medicine and Rehabilitation, Foundation Don Carlo Gnocchi, Milan, ITA; 2 Anesthesiology/Pain Medicine, H. Lee Moffitt Cancer Center and Research Institute, Tampa, USA; 3 Osteopathy, Body Lab Clinica di Osteopatia, Milan, ITA

**Keywords:** osteopathic, osteopathy, quantum physics, entanglement, biology, fascial system, myofascial, fascintegrity, fascia

## Abstract

The Foundation of Osteopathic Research and Clinical Endorsement (FORCE) is an organization that includes various figures involved in clinical and non-profit research and does not depend on any private or government body. To better understand how the human body behaves, we need to observe cellular behavior. Considering the human body as layers, districts, and regions, or just as a machine, is severely limiting to understanding the systemic mechanisms that are implemented to maintain bodily health. For some years, FORCE has contributed several articles to the literature to support the view of a human body as a unit, a fascial continuum (solid and fluid fascia) capable of interacting consciously, and not as a passive mirror, with respect to external stresses. The article reviews the tensegrity theory applied to the cell, trying to bring to light that the mechanistic vision on which this theory is based does not meet biological reality. We review some concepts related to biology, the science that studies life, and quantum physics, the science that studies the invisible physical phenomena that underlie life. Understanding that the cells and tissues are aware of the therapeutic approaches they receive could better guide the decisions of the osteopathic clinician.

## Introduction and background

The biological view of cellular behavior and the ability to communicate between cells has changed under the lens of quantum physics [1]. Biological phenomena exist as different, complex, and contemporaneous actions under the epigenetic and functional aspect, that is, the sum of multiple probabilities and chronological events in the face of external stresses [1]. Quantum phenomena are correctly applicable to observe cellular responses, through quantum coherence (equal behavior between different cells) and entanglement (distant correspondence without direct physical contact between different cells) [1].

The resistance to changing perspectives in understanding biological behavior is given by macroscopic imprinting, where a cell is not only part of the entire organism but can influence the entire organism; furthermore, the observation of a body area allows us to understand the behavior of the whole [1]. Translated into osteopathic medicine (OM), from the evaluation of a body region, it is possible to understand where one or more problems that cause symptom(s) could arise, and above all, treating a body area means translating an external stimulus into a systemic self-healthy act [2].

Based on these concepts, the cell adapts not only thanks to its own genetics but also thanks to information coming from the outside; the environment influences and can determine the function of the cell [3,4]. Translated into an OM vision, the manual approach could provide profound information to the patient's tissues, bypassing the genomic limit which, at that given moment, is present in the cells that form the tissues and cause the symptom. An epiphenomenon (manual touch) could cause genomic adaptations (epigenetic phenomena or self-healing) [1]. Each solid structure of the cell produces electromagnetic oscillations, which move (waves) towards other nearby and distant cells. This mechanism determines the alteration of the electromagnetic status of the cells involved, with the deformation of solid structures and movement of fluids (mechanotransduction) [2]. We can strongly hypothesize that this mechanism is one of the reasons behind the osteopathic therapeutic response.

Each cell demonstrates functional awareness of its own identity and collaborates with other cells to maintain the shape of tissues [1,5,6]. Each cell can behave as a single entity or as a complex network; the ability to process information creates different choices and behaviors [1]. Bringing this concept back to the clinic, the mutual reciprocity between external information and the inside of the cell strongly allows us to hypothesize that the osteopath's hand can affect the touch towards the cellular DNA. Following the concept of quantum physics, entanglement, when contact is created between one cell and another, the bond is forever [2]. This adaptation allows instant communication between cells, independent of the neural network [2].

Quantum coherence and the phenomenon of entanglement allow information to be kept in constant movement, involving cells/tissues in a state of continuous reciprocity; this determines the single identity and the formation of the contemporary identity of the tissue network [1]. This biological quantum system determines life [7,8]. The cell, with respect to the laws of thermodynamics, does not reach equilibrium, but constant entropy in a self-organizing process [9]. We could hypothesize that this self-organization is the biological process that underlies self-healing.

The cytoskeleton itself depends on quantum phenomena [1,10]. Quantum coherence allows the cell to react and to respond to the information it receives, that is, this choice is the cell's awareness [1]. This means that the cell does not respond like a mirror, it is not a simple reflection, and it does not follow a logic predetermined by a mechanical scheme: the cell is aware [1,11]. The cell cannot fit into a purely mechanical vision of tensegrity/biotensegrity. It is necessary to review and rethink how to frame biological phenomena, to try to understand what happens to the patient with the osteopathic approach. The article reviews the concept of fascintegrity, a relatively recent perspective that is based on the concept of fascial continuity, fluid and solid, where the cell is not just a passive structure but an active and conscious participant in the interaction between the patient and clinician.

## Review

History of tensegrity and biotensegrity

The term "tensegrity" (tensional integrity) was conceived by a designer and systems theorist R. Buckminster Fuller in 1960, to designate an architectural feature, where a solid structure can manage variations in tension, without changing the original characteristics [12]. The mechanical concept underlying the word is an architecture with continuous tension and with discontinuous compression [12]. The idea had its inspiration from a sculpture (called X-Piece) by the artist Kenneth Snelson in 1948 [13].

In 1977, Dr. Robbie was the first to transport the word tensegrity into the biological sphere, to try to understand the behavior of the spine and muscles, equating an architectural structure with the living [12].

Also, in the 1970s and always observing sculptures, Dr. Ingber tried to connect the concept of tensegrity to the mechanical behavior of the cell, with scientific works published in 1985 [12,13]. He theorized that the proteins of the cytoskeleton or microtubules would behave as structures in continuous tension or pre-stress, while the actomyosin complex would act as the component in discontinuous compression [12]. Currently, Ingber himself underlines how his vision is a mechanobiological theory [14].

At the 34th Annual Conference on Engineering in Medicine and Biology (1981), Dr. Levine presented a poster where he coined a new terminology, namely, the union of the word tensegrity with the word biology: biotensegrity. In this mechanistic vision, bone tissue is considered as the body region in discontinuous mechanical tension, whereas muscles and joints are considered as structures in constant tension [12].

**Figure 1:**
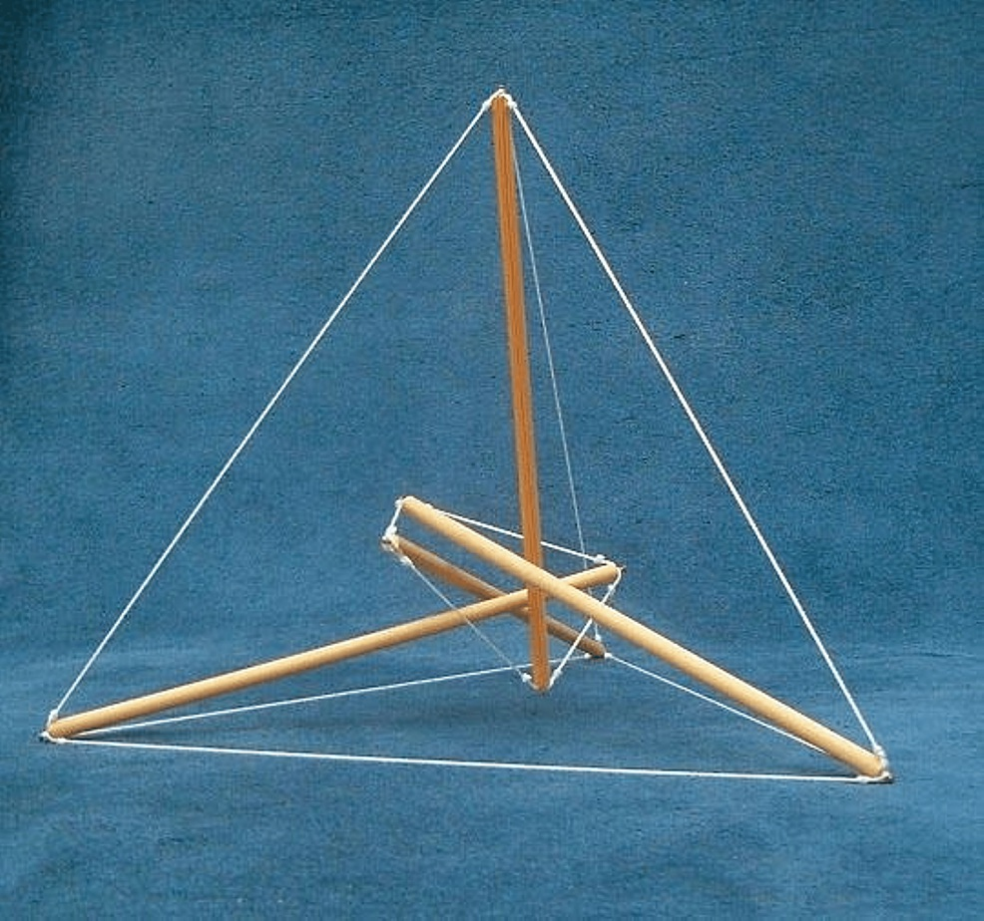
The image represents the concept of tensegrity, a rigid and architectural structure, where the wires represent the pre-stressed components, while the wood represents the discontinuous mechanical tension. The shape in this way conceived allows for continuous integrity to be preserved. Image Credit: Bruno Bordoni

The vision of tensegrity/biotensegrity is because a mechanical stimulus that the cell undergoes (compression, stretching, and more) is transmitted to the cell nucleus, via the continuous relationship of the different proteins that constitute the cytoskeleton (and the extracellular matrix), allowing the phenomenon of mechanotransduction [12,13]. This denotes a mechanical passivity, where the cell is not functionally aware, but responds like a mirror, without choosing how to respond, without measuring (Figure 1).

We agree on the notion that the body/fascial system (solid and fluid fascia) is based on the constant continuity of its components and that each structure can influence each region of the body in a biunivocal manner, from the cell to the tissue and vice versa. However, a consensus is lacking as a research group (Foundation of Osteopathic Research and Clinical Endorsement (FORCE)), in the consideration of the cell as a passive biological organization.

Mechanical models of the cell

In 2019, FORCE coined a new term to imply that the cell is not a passive living structure, but a conscious one with the ability to store information: fascintegrity [15]. As with the concept of tensegrity/biotensegrity, the concept of fascintegrity is also one of those theories to be validated.

The function of the cell is not the mathematical response to external stimuli [11]. The mechanistic vision alone is not congruent with life [11].

One of the models that tries to explain the behavior and organization of the cell is the fluid mosaic model (FMM), created in 1968 by Dr. Bothorel, Dr. Singer, and Dr. Nicholson [11]. FMM considers the membrane as a disordered conglomerate of proteins (transmembrane proteins, proteins dislocated not throughout the membrane or semi-integral, and proteins that exit the membrane or peripheral proteins), and lipids formed in a bilayer, in an environment rich in fluids [11]. The goal of the cell is to maintain its internal autonomy by keeping intracellular entropy low. The model explains the motivation for the constant congruity of the structure, where proteins and lipids remain in their position, thanks to the polarities that distinguish them; the membrane molecules are amphipathic (with hydrophilic and hydrophobic characteristics), with different polarities to bind or not with water [11]. We can image the membrane as an interlocking boundary. The limitation of this model is that the structure is static, that is, it does not explain the variations in polarities that are constantly found both inside and outside the cell, just as it does not explain the multiple variations that can be created between the cell and the outside/inside [11]. Furthermore, this model does not represent a tensegrity structure, as the structure changes the position of its components, such as lipids, or the thickness of its components [11].

The cell membrane can change its thickness depending on the polar characteristics of the proteins with the fluids. Furthermore, one of the foundations of the model is that the membrane is seen as a separation from the external environment, to allow the cell to survive, maintaining its identity, despite the exchanges that occur, thanks to the membrane proteins themselves.

The mechanistic vision of this model does not consider the cell to be alive, but a non-living part of the living body [11]. The cell can adapt, to evolve; this would not happen under the aegis of a mechanical vision of biology.

Another model that follows the previous one is the membrane nanodomains (MND) (lipid microdomains or lipid rafts), which explains cellular behavior with the presence of lipids inside the membrane. Cholesterol and sphingomyelin molecules (lipid rafts) allow the membrane to be maintained in a state of fluidity, albeit in a mechanistic manner [11,16]. The cell is understood as a compartmentalization, where each molecule responds in a mirror-like manner to the biochemical information it receives [16].

The fundamental concept of tensegrity/biotensegrity focuses on the fact that the cell perceives multiple information from the membrane to the nucleus, thanks to the informational distribution of the protein, lipid (and glycolytic) components, with structures that modify the tension while maintaining the shape [17]. Mechanotransduction, that is, the mechanometabolic response to the deformations undergone, is a mechanical adaptation, which does not reflect the biological response.

The cell is "alive"

From an evolutionary point of view, the cell can evolve, managing the information it receives, through evaluations and responses, and not simply by passive adaptation [18]. Without the capacity for active evaluation, the cell, as we know it, would not exist; this vision is part of cognition-based evolution, which differs from neo-Darwinism [18]. From an osteopathic point of view, we might think that there is always an active bidirectional reciprocal relationship between the patient and the clinician; the patient does not undergo manual treatment, but through the sum of sentient cells (the body), he reacts with sentience and seeks self-healing, which is the fundamental concept of OM [19].

**Figure 2:**
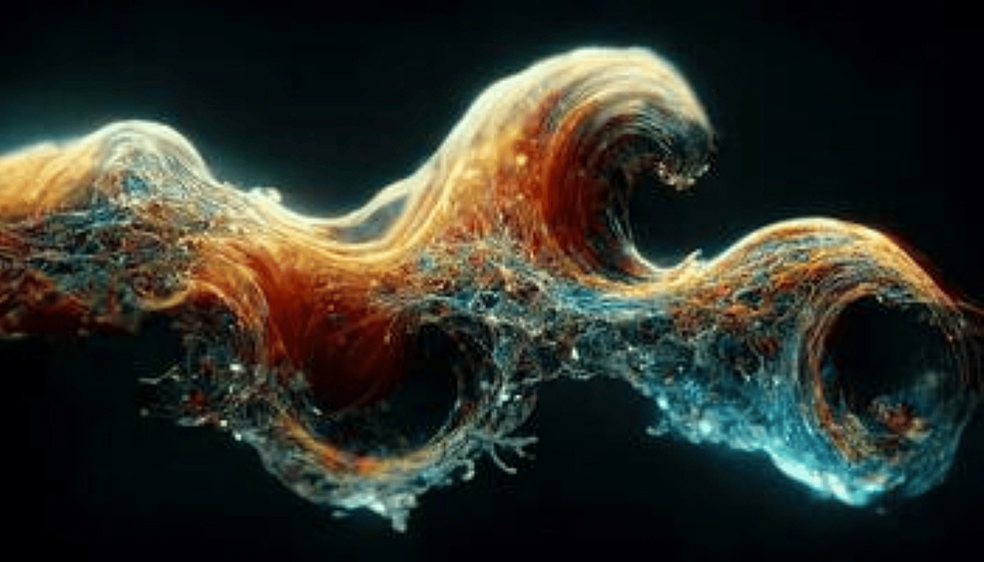
The image represents the constant movement of fluids in the three-dimensionality of living things. Cellular and extracellular fluids carry multiple biochemical and electromagnetic information, connecting the entire body at different times. Image Credit: Bruno Bordoni

Through the fascial continuum, the cell perceives the information it receives and sends, thanks to the cytoskeleton and all the structures such as ion channels, receptors, sensors, and other strategies that allow communication. These structures are considered the senses of the cell, as illustrated by the cellular basis of consciousness theory [18]. Furthermore, cellular plasma fluids are the foundation for sensory perception; the fluids allow the transmission and morphological and functional adaptation of the different structures that constitute the cell [18]. Fluids, according to the vision of this theory, are fundamental for the transport of electromagnetic signals, which make solid cellular structures capable of better processing information [18]. The cell is immersed in fluids rich in electromagnetism. Plasma could probably be the true sentient brain of the cell. Electromagnetism allows you to communicate with all cells, going beyond the mere border of the cytoskeleton and allowing adaptation; communication occurs simultaneously in the cell and between cells (Figure 2) [18].

Why do we talk about cellular awareness? The information reaching the cell is imprecise; the adequate evaluation of this ambiguous information package comes from measurement. The measurement is cognition [20,21]. The ambiguity derives from cellular subjectivity and the non-homogeneous medium through which information travels [21].

The tissue/fascial continuum derives from sentient communication between cells, with the aim of having (informational) efficiency for self-preservation [18,21]. The union of multiple cells allows the single cell to better perceive the information and manage itself: this concept is called "the wisdom of crowds" [21].

The extracellular matrix can be seen as an N-space episenome, that is, the set of information external to the cell, a library that all individual cells can access and contribute to increasing [22]. Access to the extracellular matrix (fluid tissue) allows the synchronous integration of cells into a single tissue while maintaining their homeostatic independence [22].

Communication is the result of processing; this communication always leaves a trace in the extracellular matrix [21]. Each cell communicates with nearby and distant cells, and thanks to this communication system (electromagnetism via the extracellular matrix and other communication strategies), all cells can know what is happening throughout the body [21,23]. This mechanism allows us to avoid non-physiological homeostatic fluctuations and to preventively "anticipate" non-physiological behaviors.

From the OM point of view, we could define a manual approach that is never local but always systemic and that it is possible to work a specific region of the body to simultaneously treat all body regions. This vision reconciles biology, the study of life, with the concept of fascintegrity, where the fascial continuum allows communication between all tissues and all cells in a non-mechanistic, non-stratified, and non-rigid perspective [15,24-36].

Miller et al. wrote: "Since the epicenter of cellular life is measured, a path opens to a reconciliation between quantum theory and biological expression" [21].

Behind molecular and metabolic behaviors, there are fermions (electrons, protons, neutrons) and bosons (photons, phonons), which make matter as we see it; the wave function or measurement that derives from the quantum movement would determine biology, a physical state in continuous adaptation and evolution [1,37]. According to quantum optics, the components of the cytoskeleton vibrate due to the presence and emission of biophonons (sound) and biophotons (light); this mechanism is communication/measurement that goes beyond the concept of tensegrity/biotensegrity (entanglement). The vibrational coherence that occurs in the cytoskeleton allows for immediate and whole-body bonds, enabling biological reactions [38,39].

**Figure 3:**
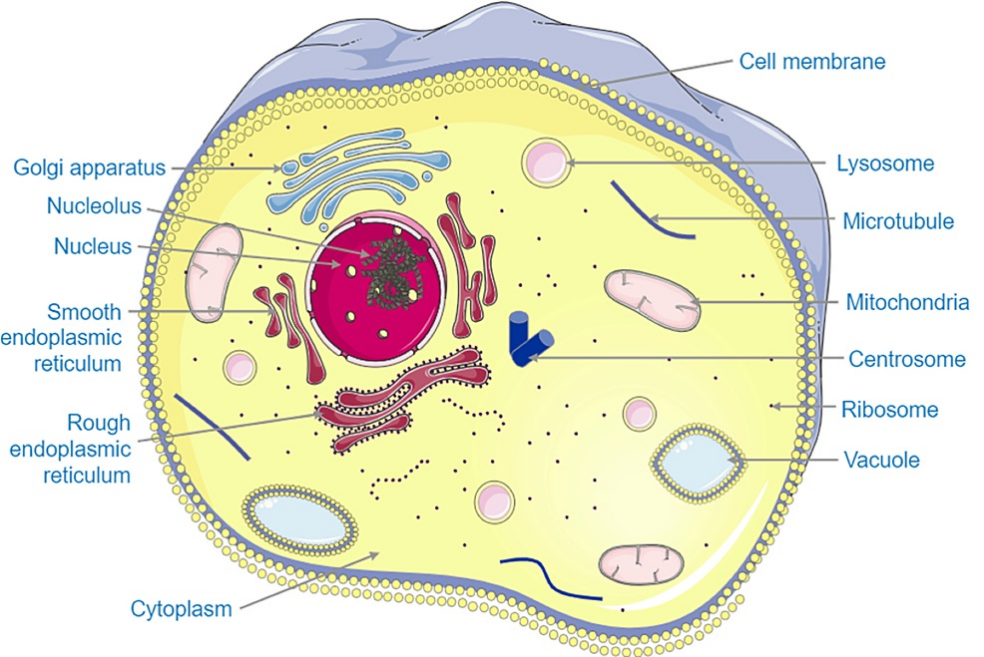
The image represents a cell, with its components: the whole cell is immersed in fluids, such as the cytoplasm and the extracellular matrix. Image Credit: Bruno Bordoni

The cell is sentient, from a biological and quantum point of view (Figure 3) [18,40,41].

Form and function

One of the tenets of OM is as follows: "structure and function are interrelated at all levels" [42]. The principle, born from the writings and teachings of the founder of OM, Still, DO, if reinterpreted considering the information that anyone can read in literature, still has a valid meaning.

According to the dictates of quantum physics, reality can change through what the observer perceives; this happens, according to the "observer theory" from the presence of entanglement, where observation can vary the behavior of electrons and change the wave function and the behavior of physical matter [2,43]. This is because observing is already an action [43]. Incorporating this notion into OM, we can strongly hypothesize the following: "Osteopathic touch is not just knowledge through palpation, but an act of mutual sharing" [2].

The cell can change its behavior, independent of the layers or macroscopic bonds via communication/information; under the aegis of physics, the form is the memory of the information received, while the expressed function is the set of communications [2]. Fascintegrity is the fascial continuum (solid and fluid) of the macroscopic expression of communication and memory [2]. The understanding of the cell drastically changes: it is no longer a mere passive structure.

The mechanistic origin of the phrase "form follows function" is from the architect Louis H. Sullivan (1896), that is, another transposition towards the biological environment by non-biological concepts, such as tensegrity [44].

Form and function, as they are perceived at a macroscopic level, are interrelated, not by a mechanical law, but by a biological and quantum condition.

Consilience and convergence

The concept of consilience is the achievement of the understanding of phenomena across different disciplines and in a faster manner [45]. It is not a mistake to observe and consider cell behavior from multiple disciplines. The term was coined by Dr. Whewell, a 19th-century British philosopher and educator, whose terminology and thought were emphasized in 1998 by Dr. Wilson, a biologist [46]. Knowledge is like a network that can be accessed in a faster way if several observers guide the search at the same time, like building a road, where more specialized figures put their knowledge to build new roads. It is a term that indicates a strong interdisciplinarity, an effective contamination between theoretical and methodological models coming from unequal fields of knowledge.

If the clinician's specialization limits the vision of the cell or does not allow interdisciplinarity, it will be difficult to fully understand the cellular function and how to put the knowledge into practice. To give an example, by combining the vision of behavioral economists and neurophysiologists, the conclusion arises that the cell/cells influence social behavior. The former think that the basis of behavioral choices that are reflected in economic choices are linked to the psyche; the latter believe that biochemical reactions are the basis of social behavior [47]. In this situation, it is the neural cells that measure and evaluate the stimuli that sense, following a choice, a biochemical and neural response that allows the final decision in the gesture [47,48]. Neurons decide which strategy is most useful for maintaining or improving their cellular structure [49,50].

Convergence is another concept that can be applied to the sciences, where achieving knowledge is a common goal of different disciplines and different research strategies [46]. Convergence becomes vital to keep up with knowledge that is emerging ever faster. Rapidly clinically identifying symptoms that apparently have no known origin requires multidisciplinary collaboration. As Roco states: "Problem-solving must go beyond a single application field, discipline, or pathway. A general problem-solving strategy for all these cases is convergence" [51].

Nobel Prize winner Sir Paul Nurse writes: "… every cell is a living entity endowed with all the properties that characterize living organisms" [52]. The fluids that are inside and outside the cell allow the dialogue of the more solid structures, which can be found inside and outside the cell. Quantum vibrations of solid structures permeate the entire body, thanks to the rapid transport of fluids. We can hypothesize that solid structures within the cell and the membrane itself would act as nanobrains (or active neural circuits), while solid structures in the extracellular matrix could act as memristors (passive nonlinear electronic components) [52]. The cell would be sentient, thanks to multiple nanobrains [53,54].

More science leads to the consilience/convergence that the cell is sentient.

Quantum biology is the new frontier of knowledge that will allow us to better understand the intricate relationships between cells, different tissues, close and distant people, and different species [55].

According to recent theories, when a cell communicates via electromagnetic oscillations, this information would behave like a holographic image projected towards other cells. This mechanism allows us to speed up the "cognitive" processes of evaluating the different information [56].

Could the pathogenesis be considered an alteration of cellular awareness? The sentient network between cells may decide to "initiate" the disease, considering that the disease could be considered a form of evolution [57].

Furthermore, what is often not taken into consideration is that the cells/atoms form matter, which matter/energy forms everything that we are and that surrounds us, including the universe [58]. Is the cell influenced by the universe or does the universe influence the cell? As osteopaths, what can we do? These are questions that await an answer from further research and studies. The questions serve not only to increase the size of the answers but to remind us to always remain humble and curious, to always adapt to the patient, and to never impose on all patients what we think we know.

To conclude, is it possible for the patient or the operator himself to create a dialogue with his own cells, seeking a way for self-healing? We hope that the next generation of researchers will be able to provide answers.

## Conclusions

To better understand how the human body behaves, we need to observe cellular behavior. A further effort is to study cellular adaptations not from a purely mechanical point of view (tensegritive/biotensegritive), but from a biological and quantum one, where the responses deriving from external stimuli denote the intelligence of the cell. Considering the human body as layers, districts, and regions, and only as a machine, is severely limiting to understanding the systemic mechanisms that are implemented to maintain bodily health. The term coined by our organization, FORCE, is to highlight that the whole body is a single continuum, where each cell is aware of what happens to other cells: fascintegrity. Finding an adequate dialogue with the cells means finding more adequate therapeutic approaches. Quantum biology is the new frontier of knowledge that will allow us to better understand the intricate relationships between cells, different tissues, close and distant people, and different species. From an osteopathic point of view, we might think that there is always an active bidirectional reciprocal relationship between the patient and the clinician; the patient does not passively or only mechanically undergo osteopathic treatment, but through the sum of sentient cells (the body), he reacts with sentience and seeks self-healing, which is the fundamental concept of OM.
